# The Growing Role of Echocardiography in Pulmonary Arterial Hypertension Risk Stratification: The Missing Piece

**DOI:** 10.3390/jcm10040619

**Published:** 2021-02-06

**Authors:** Cristiano Miotti, Silvia Papa, Giovanna Manzi, Gianmarco Scoccia, Federico Luongo, Federica Toto, Claudia Malerba, Nadia Cedrone, Susanna Sciomer, Francesco Ciciarello, Francesco Fedele, Carmine Dario Vizza, Roberto Badagliacca

**Affiliations:** 1 Department of Cardiovascular and Respiratory Sciences—Sapienza University of Rome, 00161 Rome, Italy; miotticristiano@gmail.com (C.M.); silviapapa83@gmail.com (S.P.); giovannamanzi91@gmail.com (G.M.); gianmarcoscoccia@gmail.com (G.S.); federico.luongo91@gmail.com (F.L.); federicatoto@libero.it (F.T.); claudiamalerba4@gmail.com (C.M.); susanna.sciomer@uniroma1.it (S.S.); francesco.ciciarello@uniroma1.it (F.C.); francesco.fedele@uniroma1.it (F.F.); dario.vizza@uniroma1.it (C.D.V.); 2 Internal Medicine Department, Ospedale S. Pertini, 00157 Rome, Italy; nadiacedrone@gmail.com

**Keywords:** pulmonary arterial hypertension, risk score, echocardiography

## Abstract

Pulmonary arterial hypertension (PAH) is a rare, progressive disease with a poor prognosis. The pathophysiologic model is mainly characterized by an afterload mismatch in which an increased right ventricle afterload, driven by increased pulmonary vascular resistance (PVR), leads to right heart failure. International guidelines recommend optimization of treatment based on regular risk assessments to achieve or maintain a low-risk status. Current risk scores are based on a multi-modality approach, including demographic, clinical, functional, exercise, laboratory, and hemodynamic parameters, which lack significant echocardiographic parameters. The originality of echocardiography relies on the opportunity to assess in a non-invasive way a physiologically meaningful combination of easy to measure variables tightly related to right ventricle adaptation/maladaptation to increased afterload, the main determinant of a patient’s prognosis. Echo-derived morphological and functional parameters have been investigated in PAH, proving to have prognostic relevance. Different therapeutic strategies proved to have different effects in reducing PVR. An upfront combination of drugs, including a parenteral prostacyclin, has shown to be associated with right heart reverse remodeling in a greater proportion of patients than other treatment strategies as a function of PVR reduction. Adding echocardiographic data to current risk scores would allow better identification of right ventricle (RV) adaptation in PAH patients’ follow-up. This additional information would allow better stratification of the patient, leading to optimized and personalized therapeutic management.

## 1. Strengths and Weaknesses of Current Risk Scores

Pulmonary arterial hypertension (PAH) is a rare, progressive disease with a poor prognosis. It is characterized by the remodeling of small pulmonary arterial vessels with a subsequent increase in pulmonary vascular resistance (PVR). This increase leads to right ventricular (RV) failure and ultimately to death [1]. International guidelines recommend optimization of treatment based on regular risk assessments to achieve or maintain a low-risk status [2] as defined by risk scores derived from different registries: Registry to Evaluate Early and Long-Term PAH Disease Management (REVEAL) [3], Comparative, Prospective Registry of Newly Initiated Therapies for Pulmonary Hypertension (COMPERA) [4] Swedish Pulmonary Arterial Hypertension Register (SPAHR) [5], and the French Pulmonary Hypertension Registry (FPHR) [6].

An accurate and regular risk assessment allows clinicians to follow their patients over time, update treatment goals, and identify the correct timing for lung transplantation.

REVEAL was derived from a cohort of 2716 PAH patients with all subgroups and then validated internally and externally in registries and clinical trials datasets, providing the probability of survival at 12 months in the incident and prevalent IPAH patients and in patients with associated PAH [7].

It was subsequently upgraded to REVEAL 2.0 with the modification of cut-off and relative risk points of several variables and the introduction of two further variables, including overall 13 parameters (PAH subgroups, males age > 60, renal insufficiency, New York Heart Association/World Health Organization functional class (NYHA/WHO FC), blood pressure, heart rate, all-cause hospitalization within the previous 6 months, 6 min walking distance (6MWD), brain natriuretic peptide (BNP) or N-terminal fragment of pro-BNP (NT-proBNP), pericardial effusion, % predicted diffusing capacity of the lungs for carbon monoxide, right atrial pressure (RAP), PVR). The sum of the various parameters’ risk points determines the final score that identifies the patient’s risk class.

On the other hand, European registries based their scoring system on the parameters and relative thresholds included in the European Society of Cardiology and European Respiratory Society (ESC/ ERS) guidelines.

COMPERA and SPAHR estimate the overall risk category by computing the mean of the risk grades from available variables for each patient and rounding to the nearest integer. The variables considered in the latter score are WHO FC, 6MWD, BNP or NT-proBNP, RAP, cardiac index, and mixed venous oxygen saturation. A different approach characterized the FPHR, which considered the number of low-risk variables achieved among 6MWD, WHO FC, RAP, and cardiac index, BNP or NT-proBNP.

REVEAL 1.0 and 2.0 are broadly applicable to the general PAH population, as they predict the risk in both incident and prevalent patients with various PAH etiologies. SPAHR and COMPERA were derived from incident patients with only idiopathic, heritable, drug-induced PAH or connective tissue disease-PAH. In FPHR, risk assessment was performed in incident IPAH patients.

All these scores have the advantage of being multiparametric and based on simple parameters that are easy to obtain, allowing a patient’s risk stratification into three classes (low-intermediate-high).

On the other hand, these scores have significant limitations, such as the relatively short mortality prediction period, the large number of variables needed for the REVEAL score, and the “a posteriori” nature of the validating analyses in the case of the European risk scores. Furthermore, standardization of data collection was not guaranteed in all published registries, with a significant loss of data.

All these scores are based on survival and do not consider clinical worsening and disease progression, which do not match with clinical goals to be achieved according to current guidelines [7].

Moreover, most patients end up in the intermediate-risk category, characterized by a heterogeneous population with different outcomes, representing a missed opportunity for treatment optimization and personalized medicine [8].

## 2. May Echocardiographic-Imaging Represent the Missing Piece?

The PAH pathophysiologic model is mainly characterized by an afterload mismatch, where increased RV afterload, driven by increased PVR, leads to right heart failure.

At an early stage, the RV adapts to the increased afterload to preserve stroke volume (homeometric adaptation), followed by an heterometric adaptation when the latter gets exhausted, characterized by increased filling pressures, progressive RV dilation, and hypertrophy in order to reduce wall tension (Laplace’s law), leading to eccentric hypertrophy and right heart failure (maladaptive RV). Adapted ventricles usually have preserved ventriculo-arterial coupling (a condition that occurs when the right ventricular function is adapted to the pulmonary vascular load so that energy transfer is most efficient). In contrast, maladapted ventricles usually are characterized by uncoupling of the RV to the pulmonary circulation [1,9,10].

The RV remodeling process in PAH is complex, as it results from the interaction of several factors, and the precise mechanisms determining the transition from adaptation to maladaptation and finally failure are not yet fully understood. However, what contributes most likely to these changes is the interaction between genetic factors, neurohormonal activation, etiology and duration of pulmonary hypertension over time, RV ischemia, and the amount of afterload increase [11,12,13,14].

It is now clear that patients’ clinical status and prognosis depend on the RV’s ability to adapt to the increased afterload, maintaining its function and preserving cardiac output [15,16].

The originality of echocardiography relies on the opportunity to assess a physiologically meaningful combination of easy to measure variables tightly related to RV adaptation/maladaptation to increased afterload. Echo-derived systolic functional parameters are robust though load-dependent measures of contractility, while right heart dimensional parameters reflect increased RV and RA pressure and dimensions. Thus, the RV Starling’s heterometric adaptation that comes into play in the advanced stage of the disease is easily detected by echocardiographic evaluation. Echo-derived systolic functional parameters reflect contractility adaptation and increased right heart, and inferior vena cava dimensions its failure [17]. Magnetic resonance imaging studies highlighted the importance of RV’s systolic function and dimensional parameters, showing how decreased ejection fraction and increased dimensions, despite a preserved 6MWD and decreased PVR, predicts fatal deterioration in PAH patients [18].

Echocardiography is the most user-friendly and widely used tool for assessing RV structure and function in clinical practice. It is a rapid, low-cost exam with an extremely favorable cost/benefit ratio. It may help in the non-invasive assessment of the hemodynamic RV burden in PAH, allowing a more detailed and precise analysis of the patient’s condition compared to the common clinical examination [19,20].

Considering the close correlation between ventricular morphology and function and the pathophysiological basis of PAH, it is clear that the introduction of echocardiographic parameters within the current risk scores would provide a greater opportunity to improve prognostication [21,22].

However, the initial risk scores in PAH were based on an expert clinician experience-derived multi-modality approach, in which echocardiographic parameters were limited only to RA surface area and pericardial effusion [5], or in other cases to pericardial effusion alone, as it was insufficiently available in most centers.

## 3. Echocardiographic Parameters with Prognostic Impact (Echocardiography for Prognostication in PAH)

Due to the complex and irregular right heart shape, RV and RA dimensions and their derived functional parameters—ejection fraction (EF), stroke volume, and RV fractional area change (FAC)—are all elaborated according to geometric models that approximate the RV’s shape. Nevertheless, all measurements that act as surrogates of true right heart volumes are widely used in everyday clinical practice. They are representative of the RV’s afterload mismatch condition and can help the clinician in routine patient management due to their ability to monitor disease progression and response to therapy. Studies showed that stable patients with reassuring WHO FC and 6MWD at follow-up develop a progressive deterioration of RV EF and dimensions [23]. Indeed, echo-derived morpho-dimensional and ejection phase parameters have been analyzed in PAH studies, all proving their prognostic relevance [24,25,26,27,28,29]. RA size measurements give indirect information on RV function and have prognostic value [30,31].

Dimensional RV assessment is followed by RV dysfunction evaluation defined by the echocardiographic measurement of FAC, tricuspid annular plane systolic excursion (TAPSE), tissue Doppler and myocardial strain recording of RV free wall motion, all of them representing isovolumetric and ejection phase indices of loading-induced RV pump failure. Due to its simplicity of acquisition, TAPSE, despite being a load-dependent and angle-dependent parameter reflecting mainly the lateral wall longitudinal motion, is the most popular measure used as a surrogate for RV longitudinal systolic function, with a high proven prognostic significance [28,32,33,34]. This parameter’s widespread use is also due to its high reproducibility with low inter-operator variability [35]. Pseudo normalization of TAPSE may be observed in a patient with “rocking” systolic RV motion, in which case speckle-tracking analysis may be used [36].

The ratio of TAPSE to pulmonary artery systolic pressure (TAPSE/PASP) has been validated against the ratio of end-systolic to arterial elastances (Ees/Ea) [37], which describes the ventriculoatrial coupling, and emerged as a significant independent predictor of outcome in heart failure [38] and in PAH [39]. The variation coefficient for intra and interobserver agreements is about 1% [37].

FAC is a load-dependent parameter, highly correlated with RVEF assessed on an MRI, representing the transversal component of RV contraction [40]. However, FAC has a moderate degree of intraobserver variability due to the difficulty in defining the endocardial border [31,35,41].

RV isovolumic contraction peak velocity (IVCv) and dP/dt (max) are isovolumetric phase parameters used to assess RV contractility. As all isovolumetric phase parameters, they have the advantage of being less affected by afterload. On the other hand, because of the increased RV contractility [42], these parameters usually deteriorate in the disease’s advanced stages. Significant prognostic value was observed in clinical practice for both dP/dt (max) and RV IVCv. Interobserver variability was 9.9% for dP/dt and 9% for IVCv, while intraobserver variability was 5% for IVCv [43,44].

Another prognostic relevance parameter is the RV myocardial performance index, which analyzes both RV systolic and diastolic function. However, this parameter has a high degree of inter, and intraobserver variability is load-dependent and unreliable when R-R intervals are irregular [31,41,45].

RV filling pressure can be estimated using inferior vena cava diameter and collapsibility, the presence of pericardial effusion, and the ratio between the early diastolic myocardial velocity at the lateral tricuspid annulus (E) and early diastolic tricuspid inflow (E’). All these measurements have proven to correlate with hemodynamic values, showing prognostic relevance in PAH patients [26,46,47,48].

Speckle tracking and myocardial deformation (strain and strain rate) are emerging techniques that have shown promising RV contractility evaluation results. Although regional and global 2D strains are load and angle-dependent measurements [49], several clinical studies have shown their prognostic value [50,51,52,53] with an acceptable inter and intraobserver variability.

Inhomogeneity in time to peak myocardial strain, known as RV dyssynchrony, also influences RV function, potentially to a significant extent independent from systolic function itself and showed a prognostic role in predicting clinical worsening [54,55].

The evaluation of right heart reverse remodeling (RHRR) defined in two-dimensional echocardiography by combining decreased right-sided atrial and ventricle areas and left ventricle eccentricity index, has recently emerged as a key parameter in evaluating patients’ response to therapy. Indeed, a dramatic reduction in PVR in response to therapy has been shown to be associated with RHRR, which in turn is strongly associated with improved outcomes [56,57].

However, while clinicians are becoming used to collecting echocardiographic variables for clinical practice, the most useful RV indices have been a source of debate for several years. Most echo-derived parameters lack validation in prognostic relevance, needing further investigation with dedicated multicentric prospective studies to establish solid and clinically relevant cut-off values. According to RV failure’s pathophysiologic basis in PAH, parameters like TAPSE/PASP, FAC, and RHRR represent promising parameters to improve the accuracy of current risk tools in the multi-modality assessment. Understanding the complex interactions among RV size, systolic function, and clinical parameters will require accurate and reproducible research methods that reasonably encourage collaboration between centers.

## 4. Impact of Therapeutic Strategies on Echocardiographic-Derived RV Imaging

The 6th World Symposium on PH recommends that most low- and intermediate-risk patients be treated with two oral drugs targeting NO signaling and endothelin pathways, while triple upfront therapy with parenteral prostacyclins is reserved for high-risk patients [58].

Indeed, the impact of oral monotherapies on RV morphology and function have been proven to be trivial. The BREATHE-1 echo-substudy, the only randomized trial on oral monotherapy, showed trivial beneficial effects of bosentan on echo-derived RV dimensional or systolic functional parameters [59]. Similar results have been shown in a real-life setting, where the RV end-diastolic area and FAC were found unchanged after oral monotherapy [60]. These data have been confirmed by a multicenter study with MRI in which no significant difference has been observed in RV end-diastolic volume in patients treated with oral bosentan or sildenafil [61].

Double oral therapy showed an additional effect on PVR reduction compared with oral monotherapy, with a reduction ranging between 30 and 60% [19,62,63,64]. Such modest improvement in PVR, usually <50% for the majority of patients treated with double oral therapy, is associated with a non-significative improvement in echo-determined right heart sizes, while weakly associated with improvement in RV systolic function [60]. These limited effects on RV metrics have been confirmed by the study of van de Veerdonk et al. [65], which demonstrated a mild improvement in MRI-derived RV EF, not associated with significative changes in RV end-diastolic volume.

In Sitbon et al.’s study, [66] triple upfront therapy including prostacyclins has been shown to reduce PVR by 67%, allowing a dramatic functional improvement in 6MWD and WHO FC. Similar hemodynamic improvement has been observed in the study of D’Alto et al. [67], where the combination of ambrisentan, tadalafil, and treprostinil s.c. brought significant improvement of RV dimensions and function [59,66].

Such RHRR is associated with excellent long-term survival and quality of life. However, studies showed that monotherapy or stepwise additions to initial monotherapy allow these results in a small percentage of patients (18%) [56,61,63,67]. On the other hand, an upfront combination therapy, including parenteral prostacyclins, is associated with RHRR in a greater proportion of patients than other treatment strategies as a function of PVR reduction. A sigmoid function of PVR reduction represents this relation. Indeed, it was observed that >50% of RHRR was obtained with >50% reduction in PVR [57].

Notably, in the study of D’Alto et al. [67], no significant correlation was found between changes in REVEAL Registry scores and changes in right heart dimensions, indirectly supporting the added value of simple right-sided echocardiographic imaging in PAH risk assessment.

## Data Availability

Data sharing not applicable.

## Abbreviations

6MWD	6 min walking distance
Ees/Ea	end-systolic elastance to arterial elastances
EF	ejection fraction
ESC/ERS	European Society of Cardiology and European Respiratory Society
FAC	fractional area change
IVCv	isovolumic contraction peak velocity
NT-proBNP	N-terminal pro-brain natriuretic peptide
NYHA FC	New York Heart Association functional class
PAH	Pulmonary arterial hypertension
PVR	pulmonary vascular resistance
RAP	right atrial pressure
RHRR	right heart reverse remodeling
RV	right ventricle
TAPSE	tricuspid annular plane systolic excursion
TAPSE/PASP	TAPSE to pulmonary artery systolic pressure
WHO FC	World Health Organization functional class
